# Cardiac 18F-Dopamine Positron Emission Tomography Predicts the Type of Phenoconversion of Pure Autonomic Failure

**DOI:** 10.21203/rs.3.rs-3157807/v1

**Published:** 2023-07-17

**Authors:** Abhishek Lenka, Risa Isonaka, Courtney Holmes, David S. Goldstein

**Affiliations:** Baylor College of Medicine; National Institute of Neurological Disorders and Stroke Intramural Research Program; National Institute of Neurological Disorders and Stroke Intramural Research Program; National Institute of Neurological Disorders and Stroke Intramural Research Program

**Keywords:** Pure autonomic failure, Parkinson&rsquo;s disease, multiple system atrophy, dementia with Lewy bodies, fluorodopamine

## Abstract

**Background:**

Pure autonomic failure (PAF) is a rare disease characterized clinically by neurogenic orthostatic hypotension (nOH) and biochemically by peripheral noradrenergic deficiency. Clinically diagnosed PAF can evolve (“phenoconvert”) to a central Lewy body disease (LBD, e.g., Parkinson’s disease (PD) or dementia with Lewy bodies (DLB)) or to the non-LBD synucleinopathy multiple system atrophy (MSA). We examined whether cardiac ^18^F-dopamine positron emission tomography (PET) predicts the trajectory of phenoconversion in PAF. Since cardiac ^18^F-dopamine-derived radioactivity always is decreased in LBDs with nOH and usually is normal in MSA, we hypothesized that PAF patients with low cardiac ^18^F-dopamine-derived radioactivity may phenoconvert to a central LBD but do not phenoconvert to MSA.

**Methods:**

We reviewed data from all the patients seen at the National Institutes of Health Clinical Center from 1994 to 2023 with a clinical diagnosis of PAF and data about serial ^18^F-dopamine PET.

**Results:**

Twenty patients met the above criteria. Of 15 with low cardiac ^18^F-dopamine-derived radioactivity, 6 (40%) phenoconverted to PD or DLB and none to MSA. Of 5 patients with consistently normal ^18^F-dopamine PET, 4 phenoconverted to MSA, and the other at autopsy had neither a central LBD nor MSA.

**Conclusion:**

In this case series, 40% of patients with nOH and low cardiac ^18^F-dopamine-derived radioactivity phenoconverted to PD or DLB during follow-up; none phenoconverted to MSA. Cardiac ^18^F-DA PET therefore can predict the type of phenoconversion in PAF. This capability could refine eligibility criteria for entry into disease-modification trials aiming to prevent evolution of PAF to symptomatic central LBDs.

## INTRODUCTION

Pure autonomic failure (PAF), previously called idiopathic orthostatic hypotension [49], asympathicotonic orthostatic hypotension [39], and Bradbury-Eggleston syndrome [1], is a rare form of chronic autonomic failure identified by orthostatic hypotension (OH) without a known secondary cause and without clinical evidence of motor or cognitive impairment attributable to central nervous system neurodegeneration [34]. In the general population, Parkinson’s disease (PD), the most common neurodegenerative movement disorder, and dementia with Lewy bodies (DLB), the second most common cause of dementia, occur far more frequently than does PAF. Prospective [35] and retrospective [3] longitudinal studies have found, however, that PAF diagnosed based on clinical criteria can evolve (“phenoconvert”) to these central Lewy body diseases (LBDs) or to the non-LBD synucleinopathy multiple system atrophy (MSA).

In a multi-center prospective cohort study of 74 patients with clinically diagnosed PAF, 25 (34%) phenoconverted to PD, DLB, or MSA by 4 years of follow-up [35]. A similar rate of phenoconversion (32%) was reported in an Italian cohort of 50 patients with idiopathic autonomic failure after a median of 7 years [14]. A retrospective study of 275 PAF patients evaluated at the Mayo Clinic from 2001 to 2011 reported phenoconversion in 24% to a synucleinopathy with motor or cognitive involvement [3].

Two histopathologically distinct forms of synucleinopathy have been described. LBDs such as PD and DLB entail increased deposition of the protein alpha-synuclein (aS) in neurons and nerve fibers (Lewy bodies and Lewy neurites) [45, 46], whereas MSA involves aS deposition in glial cytoplasmic inclusions [47]. These differences based on post-mortem analyses seem to have in vivo parallels, in that in LBDs there is increased deposition of native or phosphorylated aS in sympathetic noradrenergic nerves in skin biopsies [7, 31], while in MSA there can be increased deposition of aS in non-neuronal Schwann cells [10].

The LB forms of synucleinopathy (PD and DLB) differ from the non-LB form (MSA) in terms of genetics, symptomatic manifestations, progression, management, prognosis, and neuropathology [2, 6, 23, 32, 38, 47, 48]. Accurate prediction of the future phenotype therefore has important implications for clinical diagnosis and treatment and for research. Although retrospective studies have reported an association of certain features such as rapid eye movement (REM) sleep behavior disorder (RBD), bladder symptoms, stridor, and higher supine plasma norepinephrine levels with phenoconversion to MSA [14], these features may not be specific enough to predict phenoconversion at an individual level.

In a retrospective longitudinal study from the Mayo Clinic, patients who phenoconverted to MSA were reported to have had high levels of neurofilament light chain (NfL) in the cerebrospinal fluid (CSF) [43]. In a recent prospective study, protein misfolding cyclic amplification (PMCA) detected aS oligomers in the CSF of PAF patients, establishing that PAF entails central neural synucleinopathy [44]. In that study both increased levels of NfL and magnitudes of aS PMCA were associated with phenoconversion of clinically diagnosed PAF to MSA over about 2.5 years of follow-up. In the above-mentioned multi-center study [35] phenoconversion from clinically diagnosed PAF to MSA occurred during the first 2 years of follow-up, whereas most of the phenoconversion to central LBDs occurred later.

Importantly, the longitudinal studies reported above involved PAF diagnosed without regard to the occurrence of sympathetic noradrenergic deficiency, which is an established hallmark of the disease [22, 49]. Using cardiac ^18^F-dopamine positron emission tomography (PET), a validated index of myocardial norepinephrine content [36], our group consistently has found that patients with LBDs have profound myocardial noradrenergic deficiency, whereas those with MSA usually have normal cardiac noradrenergic innervation [16, 37]. In a recent cross-sectional study, cardiac ^18^F-dopamine PET distinguished PD + OH from the parkinsonian form of MSA with impressive sensitivity (92%) and specificity of (96%) [37]. As cardiac ^18^F-dopamine PET has been a highly effective imaging modality for separating LBD forms of nOH from MSA in our retrospective and cross-sectional studies [18, 31], we aimed to investigate the utility of ^18^F-dopamine PET in the longitudinal assessment of PAF patients, specifically to explore if this testing modality could predict the trajectory of phenoconversion.

## METHODS

Data were reviewed from participants in protocols approved by the Institutional Review Board (IRB) of the National Institute of Neurological Disorders and Stroke (NINDS) Clinical Protocols 94N0186, 03N0004, and 18N0140). Written informed consent was obtained from all subjects prior to any research procedures. Data were analyzed in accordance with National Institutes of Health (NIH) Clinical Protocol 000490, which is a secondary research protocol approved by the NIH IRB.

We analyzed clinical and laboratory data from patients classified as PAF from 1994 to 2023 at the NIH Clinical Center. Data were evaluated about clinical, laboratory, skin biopsy, or post-mortem findings from patients who had undergone serial evaluations over years with cardiac sympathetic neuroimaging by ^18^F-dopamine PET. In most cases putamen dopaminergic neuroimaging by ^18^F-DOPA PET was also done [15].

The presence or absence of neurogenic OH (nOH) was determined based on beat-to-beat blood pressure responses to the Valsalva maneuver and orthostatic fractional increments in plasma norepinephrine levels [22, 27]. PAF was diagnosed based on the occurrence of nOH without an identified secondary cause and unassociated with clinical evidence of a movement disorder or cognitive dysfunction [13]. Data from patients with nOH in the setting of diabetes [12], amyloidosis [40], multiple myeloma [24], autoimmune autonomic ganglionopathy [17], spinal cord injury [42], or autoimmunity-associated autonomic failure with sympathetic denervation [30] were excluded, since such patients had known or suspected secondary causes. We defined “phenoconversion” as the evolution of clinical features amounting to clinical diagnoses of PD, DLB, or MSA.

^18^F-Dopamine and ^18^F-DOPA PET were conducted as reported previously [15]. The lower limit of normal for myocardial ^18^F-dopamine-derived radioactivity is 6,000 nCi-kg/cc-mCi [19], and the lower limit of normal for the putamen/occipital cortex (PUT/OCC) ratio of ^18^F-DOPA-derived radioactivity is 2.7 [20]. The aS-tyrosine hydroxylase (TH) colocalization index in skin biopsies, a validated, quantitative measure of aS deposition in catecholaminergic nerve fibers, was calculated according to previously published methodology [31]. The upper limit of normal is 1.5. Other measures of aS deposition were the aS-protein gene product 9.5 ratio in *arrector pili* muscle [29] and the intensity of aS signal in *arrector pili* muscle. For the latter measure the upper limit of normal is 2.0 [31].

### Statistics

The frequencies of PAF patients evolving to a central LBD with low vs. normal ^18^F-dopamine-derived radioactivity were compared by Fisher’s exact test. A p value less than 0.05 defined statistical significance.

## RESULTS

**Figure 1:**
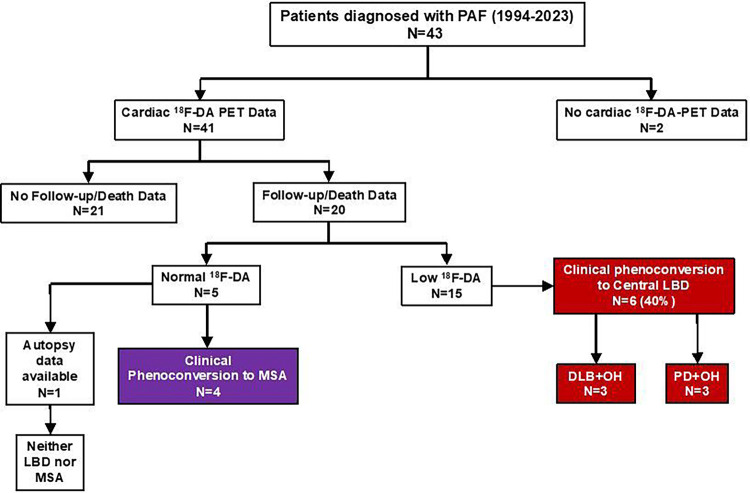
Flow diagram of patients Abbreviations: ^18^F-DA=^18^F-dopamine-derived radioactivity; DLB=dementia with Lewy bodies; LBD=Lewy body disease; MSA=multiple system atrophy; PAF=pure autonomic failure; PD+OH=Parkinson’s disease with orthostatic hypotension; PET=positron emission tomography.

Since 1994 we identified 41 patients who had nOH, did not have any of the exclusion criteria, and had available ^18^F-dopamine PET data (Fig. 1). Of the 41, 20 had clinical follow-up or death data, 15 with low ^18^F-dopamine-derived radioactivity and 5 with normal radioactivity. Among the 15 patients with low ^18^F-dopamine-derived radioactivity, 6 (40%) phenoconverted during follow-up, all 6 to a central LBD. Among the 5 patients with normal ^18^F-dopamine-derived radioactivity, 4 (80%) phenoconverted during follow-up, all 4 to MSA. Therefore, of the 20 patients with clinical follow-up or death data, 10 (50%) phenoconverted to a synucleinopathy with motor or cognitive involvement. The frequency of PAF patients with low radioactivity who phenoconverted to a central LBD (6 of 6 (100%)) differed significantly from the frequency of patients with normal radioactivity who phenoconverted to a central LBD (0 of 4 (0%); p = 0.0048).

A patient with normal ^18^F-dopamine-derived radioactivity retained a PAF phenotype until his sudden, unexpected death at 61 years old. The patient did not have post-mortem histopathological findings of either a LBD or MSA [30]. Four PAF patients phenoconverted to the parkinsonian form of MSA, based on typical clinical features of parkinsonism and low PUT/OCC ratios of ^18^F-DOPA-derived radioactivity when last seen. Of the 4, 1 is still alive with advanced disease. The other 3 died at 64, 67, and 55 years old, with the times from last evaluation to death being 3.5, 1.0, and 1.1 years. None of the MSA patients in this series had autopsy confirmation of MSA.

**Figure 2:**
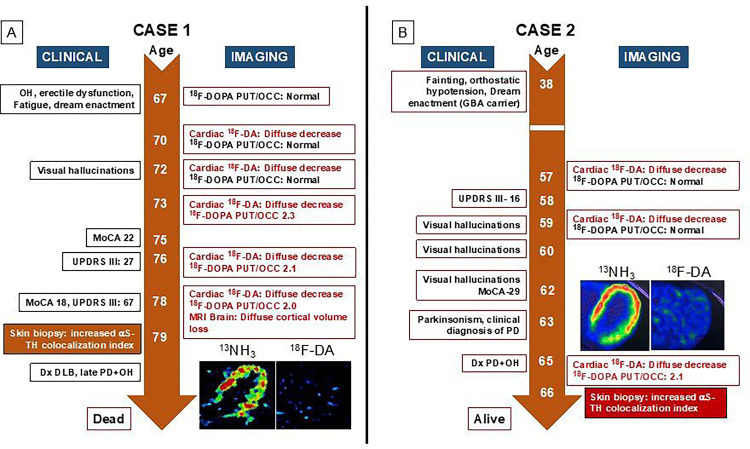
Clinical and imaging timelines of patients with pure autonomic failure (PAF) evolving to central Lewy body diseases Insets show ^13^N-ammonia and ^18^F-dopamine positron emission tomographic images, with matched spectral scales (in (A) minimum radioactivity black, maximum red; (B) minimum radioactivity black, maximum white). Abbreviations: ^18^F-DA=^18^F-dopamine-derived radioactivity; Dx=diagnosis; GBA=glucocerebrosidase gene; MoCA=Montreal Cognitive Assessment; NE=norepinephrine; ^13^NH_3_=^13^N-ammonia; OH=orthostatic hypotension; PD=Parkinson’s disease; PUT/OCC=putamen/occipital cortex ratio of ^18^F-DOPA-derived radioactivity; TH=tyrosine hydroxylase; UPDRS=Unified Parkinson Disease Rating Scale.

**Figure 3:**
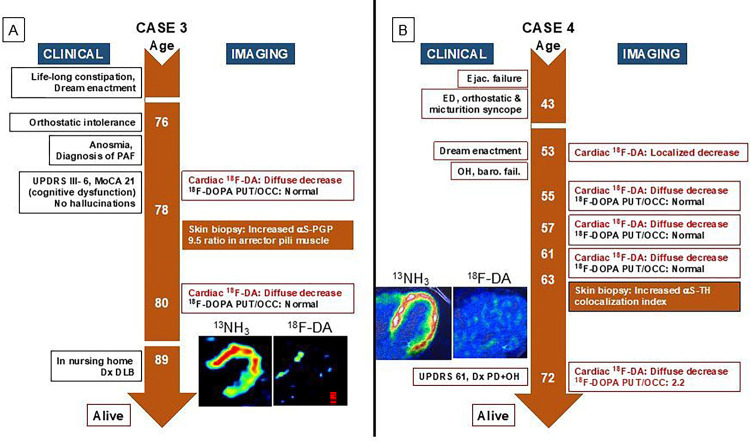
Clinical and imaging timelines of patients with pure autonomic failure (PAF) evolving to central Lewy body diseases Insets show ^13^N-ammonia and ^18^F-dopamine positron emission tomographic images, with matched spectral scales (in (A) minimum radioactivity black, maximum red; in (B) minimum radioactivity black, maximum white). Abbreviations: ^18^F-DA=^18^F-dopamine-derived radioactivity; DLB=dementia with Lewy bodies; Dx=diagnosis; ED=erectile dysfunction; Ejac. Failure=ejaculatory failure; MoCA=Montreal Cognitive Assessment; NE=norepinephrine; ^13^NH_3_=^13^N-ammonia; OH=orthostatic hypotension; PAF=pure autonomic failure; PD=Parkinson’s disease; PGP=protein gene product; PUT/OCC=putamen/occipital cortex ratio of ^18^F-DOPA-derived radioactivity; TH=tyrosine hydroxylase; UPDRS=Unified Parkinson Disease Rating Scale; UPSIT=University of Pennsylvania Smell Identification Test

**Figure 4:**
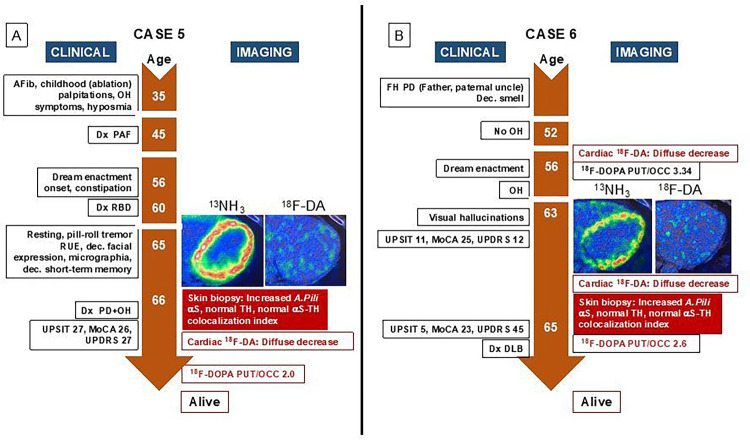
Clinical and imaging timelines of patients with pure autonomic failure (PAF) who evolved to (A) Parkinson’s disease with orthostatic hypotension (PD+OH) or (B) dementia with Lewy bodies (DLB). Insets show ^13^N-ammonia and ^18^F-dopamine positron emission tomographic images, with matched spectral scales (minimum radioactivity black, maximum white or red). Abbreviations: AFib=atrial fibrillation; *A.Pili=arrector pili* muscle; DLB=dementia with Lewy bodies; Dx=diagnosis; ^18^F-DA=^18^F-dopamine-derived radioactivity; MoCA=Montreal Cognitive Assessment; ^13^NH_3_=^13^N-ammonia; OH=orthostatic hypotension; PD=Parkinson’s disease; PUT/OCC=putamen/occipital cortex ratio of ^18^F-DOPA-derived radioactivity; RUE=right upper extremity; RBD=rapid eye movement behavior disorder; TH=tyrosine hydroxylase; UPDRS=Unified Parkinson Disease Rating Scale; UPSIT=University of Pennsylvania Smell Identification Test; y.o.=years old

**Figure 5:**
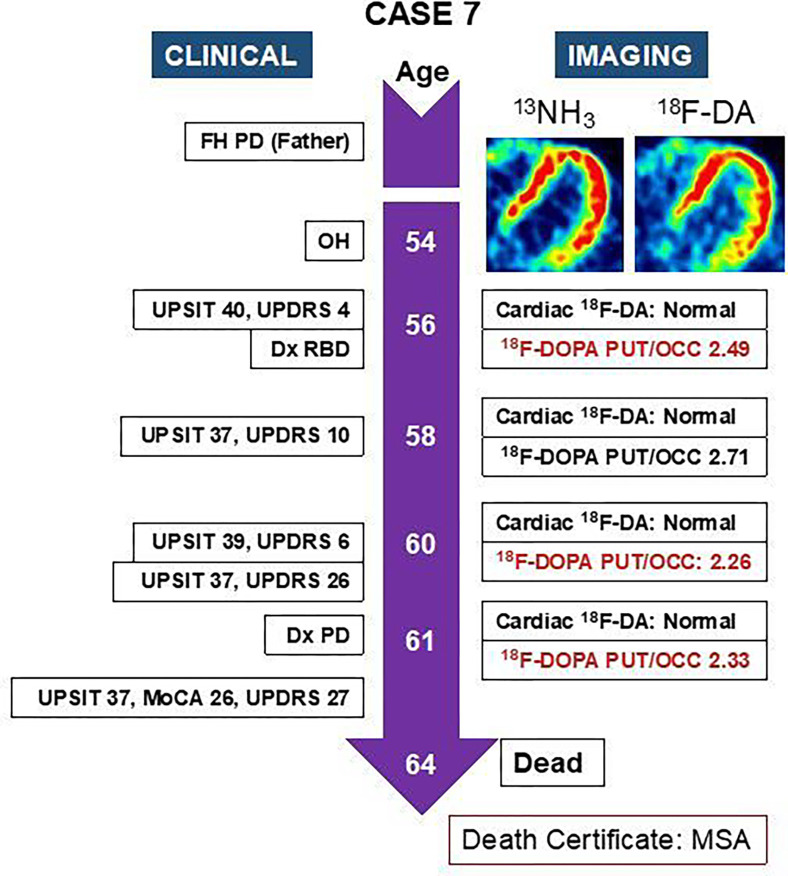
Clinical and imaging timelines of a patient with pure autonomic failure (PAF) who evolved to the parkinsonian form of multiple system atrophy (MSA). Insets show ^13^N-ammonia and ^18^F-dopamine positron emission tomographic images, with matched spectral scales (minimum radioactivity black, maximum red). Abbreviations: ^18^F-DA=^18^F-dopamine-derived radioactivity; Dx=diagnosis; MoCA=Montreal Cognitive Assessment; ^13^NH_3_=^13^N-ammonia; OH=orthostatic hypotension; PD=Parkinson’s disease; PUT/OCC=putamen/occipital cortex ratio of ^18^F-DOPA-derived radioactivity; RBD=rapid eye movement behavior disorder; UPDRS=Unified Parkinson Disease Rating Scale; UPSIT=University of Pennsylvania Smell Identification Test. Note persistently normal UPSIT scores and ^18^F-DA-derived radioactivity.

Below and in Figs. 2–5 we summarize the clinical laboratory findings of the 6 PAF patients who phenoconverted to a central LBD. We also describe one of the PAF patients who had normal cardiac ^18^F-dopamine-derived radioactivity and phenoconverted to MSA.

As noted below or in Figs. 2–5, all the PAF patients who phenoconverted to a central LBD had evidence of increased aS deposition in sympathetic noradrenergically innervated structures in skin biopsies.

### Case 1: PAF to DLB + OH (Fig. A)

This man reported OH, erectile dysfunction, and fatigue with onset at about 67 years old, in the setting of a long history of post-traumatic stress disorder and dream enactment behavior. Cardiac ^18^F-dopamine PET scans at 70, 72, 73, 76, and 78 years all demonstrated decreased ^18^F-dopamine-derived radioactivity diffusely in the left ventricular myocardium. At the time of initial evaluation at 70 years old and 2 years later, the PUT/OCC ratios of ^18^F-DOPA-derived radioactivity were normal. The first neuroimaging evidence of putamen dopamine deficiency was obtained at 73 years old (6 years after the onset of OH, 3 years after abnormal cardiac ^18^F-dopamine PET scanning). Low PUT/OCC ratios were found also in 2 subsequent scans at ages 76 and 78 years old. He first reported visual hallucinations at 72 years old, and these persisted throughout the remainder of the clinical course. He had progressive cognitive decline (Montreal Cognitive Assessment (MoCA) scores of 22 at age 75 years and 18 at age 78 years) associated with magnetic resonance image evidence of enlargement of the lateral ventricles and diffuse cortical atrophy. He had gradually progressive parkinsonism (Unified Parkinson Disease Rating Scale (UPDRS) score of 27 at age 76 years old, 67 at 78 years old). UPSIT scores consistently indicated anosmia at 70, 72, 73, and 78 years old. He died at 79 years old with a diagnosis of DLB. Autopsy revealed brainstem LBs without cortical LBs, decreased putamen dopamine content, and markedly decreased myocardial norepinephrine. αS fibrils were noted in both the sympathetic chain and apical myocardium.

### Case 2: PAF to PD + OH (Fig. 2B)

This woman reported orthostatic intolerance and dream enactment behavior since she was 38 years old. During her initial evaluation at the NIH Clinical Center at 57 years old, cardiac ^18^F-dopamine PET scanning showed decreased ^18^F-dopamine-derived radioactivity diffusely in the left ventricular myocardium. This abnormality persisted in 4 subsequent ^18^F-dopamine PET studies at ages 59, 60, 61, 63, and 65 years old. Her 2 ^18^F-DOPA PET scans (at 57 and 59 years old) were normal. The first evidence of a ^18^F-DOPA PET abnormality occurred at 60 years old, 3 years after the first abnormal cardiac ^18^F-dopamine PET scan. Visual hallucinations began at 59 years old and persisted. At 65 years old her UPDRS score was 58, and she was diagnosed clinically with PD. She did not have cognitive dysfunction as indicated by her MoCA. UPSIT scores at 58, 61, 63, and 65 years old showed anosmia, and a skin biopsy at 65 years old demonstrated an increased αS-TH colocalization index. As of this writing she is alive at 66 years old and carries a diagnosis of PD + OH.

### Case 3: PAF to DLB + OH (Fig. A)

This woman reported constipation and dream enactment behavior for as long as she could remember. When first evaluated at the NIH Clinical Center at 76 years old she reported orthostatic intolerance and lack of sense of smell. She had nOH, decreased ^18^F-dopamine-derived radioactivity diffusely in the left ventricular myocardium, normal putamen ^18^F-DOPA-derived radioactivity, normal CSF DOPA and 3,4-dihydroxyphenylacetic acid (DOPAC) levels [26], and increased αS signal in arrector pili muscle in a skin biopsy [31]; she was diagnosed with the LB form of PAF. About 1–2 years later she reported decreased cognitive function, and at 77 years old her MoCA score was 22. Follow-up PET neuroimaging showed low cardiac ^18^F-dopamine-derived radioactivity, while the PUT/OCC ratio of ^18^F-DOPA was normal. As of this report she is alive at 89 years old but is institutionalized with advanced DLB.

### Case 4: PAF to PD + OH (Fig. 3B)

This man reported dream enactment behavior and ejaculatory dysfunction since he was 39 years old. About 2 years later he developed erectile dysfunction, which persisted. About this time he had an episode of micturition syncope. Upon initial evaluation at the NIH Clinical Center at 48 years old he had decreased ^18^F-dopamine-derived radioactivity in the left ventricular free wall, but interventricular septal radioactivity was normal. Subsequent ^18^F-dopamine PET scans at 50, 52, and 59 years old demonstrated decreased radioactivity throughout the left ventricular myocardium. Skin biopsy at 56 years old showed an increased αS-TH colocalization index. His initial ^18^F-DOPA PET scan at 53 years old revealed a decreased PUT/OCC ratio of ^18^F-DOPA-derived radioactivity (2.31), but subsequent scans at 55, 57, 61, and 63 years old disclosed normal PUT/OCC ratios. After 19 years of follow up, at age 72 years, he developed parkinsonian symptoms, had a UPDRS score of 61, and had a low PUT/OCC ratio at 2.2. He is alive with a diagnosis of PD + OH.

### Case 5: PAF to PD + OH (Fig. A)

This woman developed orthostatic intolerance, palpitations, and impaired sense of smell at 35 years old. She was diagnosed with PAF at the Mayo Clinic at 51 years old. She subsequently developed dream enactment behavior, and RBD was diagnosed when she was 52 years old. She did not have any motor symptoms or cognitive dysfunction until she was 60 years old. At age 61 years she developed a resting tremor of the hands, micrographia, and hypomimia. Her cardiac ^18^F-dopamine PET in 2021 (age 60 years) revealed diffusely decreased radioactivity in the left ventricular myocardium. ^18^F-DOPA PET in 2022 (age 61 years) showed a decreased PUT/OCC ratio of 2.2 [15]. As of this report she has not developed visual hallucinations or cognitive dysfunction. Based on these data she is deemed to have phenoconverted to PD + OH.

### Case 6: PAF to DLB + OH (Fig. B)

This man reported decreased sense of smell for many years. His father had been diagnosed with PD at 55 years old and had died at 72 years old. The patient lost consciousness in about 2009–2010 at an outdoor church service when he got up to go to the bathroom. When first seen at the NIH in 2011 at 52 years old he did not have OH. Subsequently he developed episodic OH that became persistent and consistent. Dream enactment was first noted when he was about 56 years old. He developed constipation and in 2020 required disimpaction in an emergency room. In 2021, at 62 years old, he had to retire due to cognitive decline. He lost substantial weight because of decreased appetite. In 2023 he reported the onset of episodic visual hallucinations, brain fog, decreased memory, whispery voice, sloppy handwriting without a change in size, and slow movements with all activities. His MoCA score declined from 25 in 2021 to 23 in 2023. Cardiac ^18^F-dopamine PET in 2021 and 2023 showed diffusely decreased radioactivity in the left ventricular myocardium. From 2021 to 2023 the PUT/OCC ratio of ^18^F-DOPA-derived radioactivity decreased from within normal limits at 3.34 to subnormal at 2.60. He is alive with a diagnosis of DLB.

### Case 7: PAF to MSA (Fig. 5)

This woman had dream enactment behavior (RBD diagnosed by polysomnography), constipation, urinary incontinence, heat intolerance, and chronic orthostatic, post-prandial, and post-exertional dizziness. Her father had had PD. Upon initial evaluation at 56 years old she had nOH, normal olfaction (UPSIT score 40), a decreased PUT/OCC ratio of ^18^F-DOPA-derived radioactivity at 2.49, and normal cardiac ^18^F-dopamine-derived radioactivity. Subsequent ^18^F-dopamine PET and UPSIT scores were consistently normal. At 61 years old she had a UPDRS score of 26 and a low PUT/OCC ratio at 2.33. Based on neurological examination at that time by a Board-certified neurologist specializing in movement disorders, she was diagnosed with PD. She died 3 years later. According to the obituary and the Certificate of Death from the Vital Records Department of her county of residence, she had died of MSA. There was no autopsy.

## DISCUSSION

In this study we reviewed longitudinal follow-up data from PAF patients who had undergone comprehensive clinical laboratory testing including cardiac ^18^F-dopamine PET and during follow-up phenoconverted to a symptomatic central synucleinopathy. Six patients phenoconverted to a central LBD; all 6 had low ^18^F-dopamine-derived radioactivity initially or during follow-up. Four patients phenoconverted to MSA; all 4 had persistently normal ^18^F-dopamine-derived radioactivity. Previous cross-sectional studies have shown that among patients with nOH those with central LBDs have low ^18^F-dopamine-derived radioactivity, whereas those with MSA usually have normal radioactivity [16, 18, 25, 37]. Here we report longitudinal data supporting the view that in PAF low ^18^F-dopamine-derived radioactivity predicts phenoconversion to a central LBD and not to MSA.

All the PAF patients who phenoconverted to a central LBD also had evidence for increased aS deposition in sympathetic noradrenergic nerves in skin biopsies, another aspect that separates central LBDs from MSA [8–10]. Whether elevated aS in sympathetic nerves predicts the pattern of phenoconversion of PAF remains unknown.

Based on previous reports, the type of phenoconversion (to a central LBD or to MSA) depends importantly on the duration of follow-up. The longer PAF patients are followed, the greater the prevalence of phenoconversion to a central LBD [35]. Since the disease process in MSA progresses quickly [23], the latency of phenoconversion from PAF to MSA is relatively short. Rapid progression of MSA can explain the relatively small number of patients in the present study who phenoconverted to MSA, since such patients would be unlikely to return for follow-up testing.

Similar to PAF, patients with isolated RBD are known to be at risk for phenoconversion to a central synucleinopathy [4]. A recently published study used cardiac ^123^I-metaiodobenzylguanidine (^123^I-MIBG) scintigraphy, which is analogous to cardiac ^18^F-dopamine PET, for predicting phenoconversion of patients with isolated RBD [41]. In that study RBD patients who phenoconverted to a central LBD had significantly lower heart-to-mediastinum ratios of ^123^I-MIBG-derived radioactivity than did other RBD patients.

In the present study there was a wide range of latency from the diagnosis of PAF to the diagnosis of a central LBD, from about 3 years to about 19 years of follow-up. A key task for the future is to develop means to identify when the disease process is transitioning from a peripheral to a central LBD. One possibly informative biomarker is ^18^F-DOPA PET, since patients with PAF have normal PUT/OCC ratios of ^18^F-DOPA-derived radioactivity, whereas patients with PD have low PUT/OCC ratios [21]. PAF also entails normal CSF levels of DOPAC, the main neuronal metabolite of dopamine, whereas in PD and MSA CSF DOPAC is subnormal [26]. Under the presumption of an ascending spatio-temporal process within the brainstem with earlier involvement of the pontine locus ceruleus than of the midbrain substantia nigra [5, 28], neuromelanin magnetic resonance imaging might also help identify PAF evolving to a central LBD [11]. As of this writing, no studies have reported on whether assays of NfL or aS seeding identify the timing of phenoconversion from PAF to a central LBD vs. MSA. Future studies should also attempt to identify factors that might distinguish phenoconversion to PD from phenoconversion to DLB [33].

Although 40% of our patients with nOH and low ^18^F-dopamine-derived radioactivity phenoconverted to a central LBD, 60% were lost to follow-up or are still being followed. The 40% figure therefore is likely to be an underestimate.

### Limitations

The number of patients reported here was small. PAF and MSA are rare disorders, and following such patients longitudinally using sophisticated objective measures over years requires substantial time, effort, and expense. Nevertheless, the clinical and laboratory findings of the patients described here support the utility of cardiac ^18^F-dopamine PET for predicting the phenoconversion trajectory of PAF.

^18^F-Dopamine PET is available only at the NIH Clinical Center, which makes it difficult to replicate the findings. ^123^I-MIBG scintigraphy is widely available but is not FDA approved for distinguishing LB from non-LB forms of nOH. No study to date has compared ^18^F-dopamine vs. ^123^I-MIBG imaging in the same patients.

In the 4 PAF patients who phenoconverted to probable MSA there was no post-mortem neuropathologic confirmation of the diagnosis.

### Implications

The best way to gain insights into the natural course of PAF is through longitudinal studies. We illustrate in this longitudinal study that cardiac ^18^F-dopamine PET is an effective modality for predicting the trajectory of phenoconversion of PAF. This capability could facilitate enrollment into appropriate disease-modifying clinical trials.
